# Graduate Medical Education Reform: Moving the Elephant

**DOI:** 10.1007/s11606-014-2939-1

**Published:** 2014-07-01

**Authors:** Frederick J. Meyers, Mark C. Henderson

**Affiliations:** University of California Davis School of Medicine, 4610 X St. #3101, Sacramento, CA 95817 USA

Over the past decade, various graduate medical education (GME) reform proposals^1^, ^2^ have been published, but few of their recommendations have been implemented. In this issue, the Society of General Internal Medicine (SGIM) has released another sensible roadmap for GME reform, recommending increased funding for workforce assessment studies, all-payer GME financing, and increased accountability of GME institutions for producing physicians to better meet the needs of society.^3^ At a time when so many consensus statements have languished, how do we avoid a similar fate for this proposal?

We propose that the answer lies in making our academic health centers (AHCs) more accountable—by turning them into learning health care systems (LHCS), which are integrated and aligned with GME. The LHCS model, originally proposed by the Institute of Medicine, embraces patient/family-centered care and utilizes systems engineering, decision support and payment incentives to promote continuous quality improvement (QI), reduction of waste and harm, and strong community linkages that improve population health.^4^ The culture and incentives of AHCs have a tremendous influence on GME, and so must be considered if any reform effort is to be successful.

As SGIM emphasizes, we must rapidly and reliably shore up the leaky primary care (PC) pipeline. Despite widespread agreement about the importance of a robust PC workforce to improve health outcomes, the current output of GME programs is inadequate to meet demand. To address the shortage, 40-50 % of US medical graduates (USMGs) must join the PC workforce, but currently fewer than 20 % do. Some of the most prestigious AHCs graduate the lowest percentages of PC physicians.^5^ Over 90 % of students choosing family medicine enter PC practice; yet some AHCs do not even have family medicine residency programs.

Simply training more PC physicians will not be enough. USMGs do not reflect the increasing racial, ethnic, and socio-economic diversity of our communities. In California, 4 % of current physicians are Latino, compared with 40 % of the population.^6^ California’s Central Valley, the nation’s agricultural hub, suffers from extreme shortages of both PC and specialty physicians.^6^ Underrepresented minority graduates are more likely to work in underserved communities than their nonminority counterparts.^7^

To cultivate the next generation of PC physicians, we must tackle the issue of ambulatory practice redesign. In hospital-based clinics, trainees typically confront a lack of necessary resources and infrastructure to deliver high-quality, coordinated care. Yet in-patient rotations provide them the opportunity to work in multidisciplinary teams, spend time with patients, and experience a controllable lifestyle. It’s no wonder students and residents shun PC careers. AHCs must redesign their practices or deploy trainees away from the hospital to community partners who can adequately prepare PC physicians.

However, many AHCs resist reforms aimed at expanding PC and ambulatory redesign. After all, they derive significant financial benefit from the inpatient-focused GME system, in which residents provide substantial amounts of service to patients, and thus enhance hospital revenues.^8^

The book *Switch* uses a metaphor of an elephant and a rider to illustrate how difficult systemic change can be.^9^ Whenever the rational rider and the six-ton elephant disagree, the rider loses to the whims of the giant below. So knowing the right course is not sufficient to cause change. The elephant must be compelled to embark on a new path. Rather than informing the rider (GME leaders) we need to motivate the elephant (AHCs) to change direction. Otherwise, SGIM’s recommendations will go unheeded.

AHCs face extinction within the emerging high-value health system, in which they must compete by providing the highest quality care at a reasonable cost. The LHCS model provides them a lifeline. In this model, residents—who provide the majority of the care in AHCs—are actively engaged in QI, learning to become responsible stewards of our increasingly precious health care resources.^10^ If AHCs fail to change, trainees will dismiss high value care as a purely academic exercise within an environment that incentivizes wasteful diagnostic testing and imaging. This high-utilization fee-for-service paradigm has been the financial backbone of many AHCs. In a LHCS, the focus of GME would build on the evaluation of educational competencies and provide meaningful experiences in high-value care delivery and population health improvement.

So how do we get there? We would begin by actively engaging the leadership of AHCs in efforts to bolster the PC pipeline and improve the health of their local communities. Medical school deans must elevate the stature of PC within their institutions, including investing in improving the ambulatory experience for trainees. Medical schools must accurately track and report numbers of graduates entering PC practice and develop programs that reduce the cost of medical school (e.g., loan forgiveness, scholarships) for students who choose PC careers.

AHCs should respond to society’s need for high access, high quality, and high value health care. GME and AHCs must make a greater commitment to academic-community partnerships such as Teaching Health Centers and Federally Qualified Health Centers to improve care in underserved areas. These sites present an opportunity for new models that improve access, embrace inter-professional care, and integrate novel strategies for areas such as concomitant mental health and physical health care. Learners are integral to a new team that improves chronic disease management using community resources such as food banks, legal aid and housing agencies, and transportation programs for the most vulnerable. Patients, families, and community organizations become partners in a transparent, expanded LHCS.

AHC leadership must make an explicit institutional commitment to promoting a high-value, cost-conscious care curriculum for all learners.^11^ The institutional QI infrastructure should be held responsible for resident QI activities, and should be applied to the ambulatory setting with equal rigor as is done within the hospital, so that trainees learn high quality ambulatory care delivery and population health management. Intermountain Health has done this successfully with practicing physicians by designing data systems and reorganizing management structures to increase accountability and drive QI, while producing substantial cost savings.^12^ The best way to reduce cost is to improve quality.

Every year as Congress ponders the $9.5 B GME price tag for the Medicare program, the possibility of a dramatic reduction in the number of funded residency positions looms—at a time when the country needs more and better-prepared physicians. If such cuts occur, we might face the day when newly minted USMGs cannot secure residency positions.

We conclude by making a bold proposition to AHC leaders. Rather than continuing to develop product lines to maximize profit and utilizing trainees to provide mostly inpatient specialty care, let’s invest GME dollars in enabling the next generation of clinicians to learn and deliver better quality across the continuum of care including our local communities. This paradigm shift would attract support for GME from a broad coalition including patients, the public, and payers.
